# Serum Indicators Reflecting Gastric Function May Also Correlate with Other Extragastric Diseases

**DOI:** 10.1155/2015/867495

**Published:** 2015-08-03

**Authors:** Yuehua Gong, Wei Wang, Yi Li, Yuan Yuan

**Affiliations:** ^1^Department of Tumor Etiology and Screening, Cancer Institute and General Surgery, The First Affiliated Hospital of China Medical University and Key Laboratory of Cancer Etiology and Prevention, Liaoning Provincial Education Department, Shenyang 110001, China; ^2^Health Examination Center, The First Affiliated Hospital of China Medical University, 155 North Nanjing Street, Heping District, Shenyang 110001, China

## Abstract

*Aim*. Serological indicators of organ function can reveal intrinsic links between different organs. The present study aimed to determine the correlations of serum indicators for gastric and extragastric function. *Methods*. A total of 823 individuals were enrolled. Data on indicators reflecting blood lipids, blood glucose, indexes of stomach, kidney, liver, and thyroid function, and *H. pylori* IgG antibody level were collected. *Results*. As creatine (Cr) levels increased, PGI (pepsinogen I), PGII concentrations, and PGI/II ratio increased monotonically from 79.7 to 105.15 *µ*g/L, 6.5 to 8.4 *µ*g/L, and 11.97 to 12.27, respectively (*P* < 0.05). As thyroid peroxidase antibody (TPOAb) levels increased, PGI level decreased from 100.85 to 84 *µ*g/L (*P* < 0.05) and as thyroid stimulating hormone (TSH) increased, PGI/II ratio increased monotonically from 11.54 to 12.68 (*P* < 0.05). As triglyceride (TG) levels increased, gastrin 17 (G17) concentrations increased monotonically from 1.73 to 2.7 pmol/L (*P* < 0.05). As serum glucose and glycated hemoglobin (HbA1C) increased, PGI/II concentrations increased monotonically from 11.98 to 12.67 and 9.7 to 13.54 (*P* < 0.05), respectively. *Conclusions*. Serum PG and G17 levels were associated with blood glucose and lipids, kidney function, and thyroid function but not with liver function. Serum indicators reflecting gastric function may correlate not only with primary diseases, but also with other extragastric diseases.

## 1. Introduction

Serum indicators are measurable factors that reflect the normal physiological state of an organism. For example, alanine aminotransferase (ALT) and alkaline phosphatase (ALP) are biomarkers of liver function; creatinine (Cr) and urea nitrogen are biomarkers of renal function; triiodothyronine (FT3) and free thyroxine (FT4) are biomarkers of thyroid function; and high-density lipoprotein (HDL), low-density protein (LDL), glucose, and glycosylated hemoglobin (HbA1C) are related to metabolic function. Some biomarkers of gastric function, such as pepsinogen (PG), have also been recognized. PGI is produced in the stomach by the body and fundus, and PGII is produced primarily in the oxyntic gland mucosa of the stomach, the gastric antrum, and the duodenum. Several studies have found that PGI and PGII are secreted into the gastric lumen and 1% of them are also leaked into circulating blood [1, 2]. Serum PG levels seem to be related to the morphologic and functional changes in the stomach, and their use as “serological biopsy” has been reported for over 20 years [3–5]. Low serum PGI and a low PGI/PGII ratio have been recognized as useful diagnosis biomarkers for the corpus atrophic gastritis (AG) and for patients screening at high risk of gastric cancer [6–8]. Gastrin normally regulates gastric acid secretion by stimulating the proliferation of enterochromaffin-like cells and the release of histamine [9]. Serum gastrin has become an important biomarker for gastric antrum inflammation [10].

Interactions between organs are vital for allowing the body to adjust the function of each organ according to the needs of the body as a whole. Associations between serological indicators reflecting different organ functions are thus also important for revealing intrinsic links between different organs. Changes in gastric function may affect absorption, resulting in malnutrition and a consequent decline in immune function, thus increasing susceptibility to other diseases [11–13]. However, few studies have examined associations between serum indicators of gastric function, such as PGI, PGII, PGI/II ratio and gastrin-17, and extragastric functional parameters. Further investigation of these relationships will improve our understanding of the factors influencing gastric function and its correlation with gastric diseases. In this study, we investigated the correlations between serum indicators of gastric and extragastric functions, with the aim of improving our understanding of these interactions and identifying factors with potential clinical applications.

## 2. Methods

### 2.1. Study Population and Data Collection

This retrospective study was conducted during 2009 to 2012 at The First Affiliated Hospital of China Medical University, Shenyang, Liaoning Province. A total of 823 participants (518 men, 305 women; median age, 49 years; range 25–84) from the health checkup center of the hospital were enrolled in the study. All the participants have the information of PGI, PGII, gastrin-17 and blood glucose, lipids (glucose, triglyceride (TG), LDL, and HDL) and kidney function (Cr and urea nitrogen), liver function (ALT and ALP), thyroid function (FT3, FT4, TSH, TPOAb, and thyroglobulin antibody (TGAb)). However, information on HbA1C was only acquired for 57 participants.

### 2.2. Ethical Statement

The collection of data from the laboratory information system was approved by the Human Ethics Review Committee of The First Affiliated Hospital of China Medical University (Shenyang, China). Written or verbal consent was not considered necessary for the secondary use of data and because the data analyzed were deidentified.

### 2.3. Statistical Analysis

We compared the concentrations of gastric function indicators among participants grouped according to the quartiles of extragastric indicators' levels. All statistical analyses were performed with SPSS version 13.0 software (SPSS, Chicago, IL, USA). The distribution of variables was assessed by the Kolmogorov-Smirnov test. Non-normally distributed variables were analyzed using the median and interquartile range. An ordinal logistic regression model was applied to analyze the relationships between gastric function and other biomarkers among the three groups adjusted for sex, age, and* H. pylori* status. A two-sided *P* value < 0.05 was considered statistically significant.

## 3. Results

### 3.1. Baseline Characteristics of the Study Population

A total of 823 patients with serum indicators of gastric and extragastric functions were included. The distributions and normal reference ranges of the selected characteristics are shown in Table 1.

### 3.2. The Correlations between Gastric Function Parameters and Cr and Urea Nitrogen Levels in Serum

Serum PGI, PGII, PGI/II, and gastrin-17 concentrations across four Cr or urea nitrogen levels are shown in Table 2. As Cr levels increased, PGI, PGII concentrations and PGI/II ratio increased monotonically from 79.7 to 105.15 *µ*g/L, 6.5 to 8.4 *µ*g/L, and 11.97 to 12.27, respectively (*P* < 0.05). There were no significant differences in serum G17 among Cr groups (*P* > 0.05). As urea nitrogen levels increased, there were no significant differences in serum PGI, PGII, PGI/II, and G17 between groups (*P* > 0.05).

### 3.3. The Correlations between Gastric Function Parameters and ALT and ALP Levels in Serum

Serum PGI, PGII, PGI/II, and gastrin-17 concentrations across four serum ALT and ALP levels are shown in Table 3. There were no significant differences in serum PGI, PGII, PGI/II, and G17 concentrations between participants grouped according to ALT or ALP level (*P* > 0.05).

### 3.4. The Correlations between Gastric Function Parameters and T3, T4, TSH, TPOAb, and TGAb Levels in Serum

Serum PGI, PGII, PGI/II, and gastrin-17 concentrations across four serum levels of T3, T4, TSH, TPOAb, and TGAb levels are shown in Table 4. PGI level decreased from 100.85 to 84 *µ*g/L as TPOAb increased (*P* < 0.05). There were no significant differences in serum PGII, PGI/II, and G17 concentrations between participants grouped according to TPOAb level (*P* > 0.05). As TSH increased, PGI/II ratio increased monotonically from 11.54 to 12.68 (*P* < 0.05). There were no significant differences in serum PGI, PGII, and G17 concentrations between participants grouped according to TSH level (*P* > 0.05). Also there were no significant differences in serum PGI, PGII, PGI/II, and G17 concentrations between participants grouped according to T3, T4, or TGAb level.

### 3.5. The Correlations between Gastric Function Parameters and TG, HDL, and LDL Levels in Serum

Serum PGI, PGII, PGI/II, and gastrin-17 concentrations across four serum levels of TG, HDL, and LDL are shown in Table 5. As TG levels increased, G17 concentrations increased monotonically from 1.73 to 2.7 pmol/L (*P* < 0.05). There were no significant differences in serum PGI, PGII, and PGI/II concentrations between participants grouped according to TG level (*P* > 0.05). Also there were no significant differences in serum PGI, PGII, PGI/II, and G17 concentrations between participants grouped according to serum HDL and LDL levels (*P* > 0.05).

### 3.6. The Correlations between Gastric Function Parameters and Glucose and HbA1C Levels in Serum

Serum PGI, PGII, PGI/II, and G17 concentrations across four serum levels of glucose and HbA1C are shown in Table 6. As serum glucose increased, PGI/II concentrations increased monotonically from 11.98 to 12.67 (*P* < 0.05). There were no significant differences in PGI, PGII, or G17 concentrations according to glucose levels. Similarly, as HbA1C levels increased, PGI/II concentrations increased monotonically from 9.7 to 13.54 (*P* < 0.05). There were no significant differences in serum PGI, PGII, and G17 according to HbA1C levels.

## 4. Discussion

The present study explored the correlations between serum indicators of gastric function, including PGI, PGII, PGI/II, and gastrin-17, and multiple serum biomarkers of extragastric functions in a Chinese health checkup population. Our results suggest that serum PG and G17 levels were associated with kidney function, thyroid function, blood glucose, and lipids but not with liver function. To date, just one similar article had been published in a Japanese population, which investigated the relationship just only between PGI/II ratio and limited extragastric indicators including glucose, triacylglycerol, uric acid, cholinesterase, and hemoglobin [14].

Serum indicators including PGI, PGII, and G-17 may reflect the morphologic and functional changes in the stomach, and their use as “serological biopsy” for gastric diseases has been reported for over 20 years [3–5, 10]. In our preliminary study, we also found that, along with the sequence of Normal→Superficial Gastritis→Gastric Erosion and Ulcer→Atrophic Gastritis→Gastric Cancer, the serum PGI and PGII levels increased while the PGI/II ratio decreased [15]; sPGII significantly increases in diseased and* H*.* pylori*-infected stomach and is a useful biomarker to differentiate between diseased and normal stomachs [16]. However, it is still unclear whether serum indicators of gastric function also can reflect extragastric functional changes. Exploring the correlations of serum indicators for gastric and extragastric functions will contribute to a better understanding of the clinical value of these indicators.

Cr and urea nitrogen are useful and inexpensive method of evaluating renal dysfunction. A high serum Cr concentration may indicate a failure of diseased kidneys to filter Cr from the blood effectively. A previous study also found that serum PG I concentrations were elevated as the renal function declined [17]. Paimela et al. found that uremic patients displayed a gastric acid-secretion capacity within the normal range but had significantly elevated serum gastrin and PGI concentrations [18]. Nakahama et al. also reported that, in the group of chronic glomerulonephritis patients, a positive correlation between the serum Cr and the pepsinogen levels was found [19]. In the present study, we found that as Cr levels increased, PGI and PGII concentrations and PGI/II ratio increased monotonically. Most PG is usually secreted into the stomach cavity, but a small proportion enters the blood and is excreted by the kidneys [1]. We speculated that an increase in Cr concentration may result in reduced excretion of PGI and PGII from the diseased kidneys, with a consequent increase in serum PGI and PGII levels. These results suggested that elevated serum PG concentrations should consider not only the gastric dysfunction but also abnormal renal function.

An immunological association between autoimmune thyroid diseases (AITD) and autoimmune gastritis (AIG) was first suggested in the early 1960s [20]. In patients with AITD, AIG is characterized by atrophy of the corpus and fundus of the stomach and by the presence of serum autoantibodies to parietal cells and autoantibodies to intrinsic factor. Autoimmune attack of the parietal cells may cause reduced acid secretion and PG level through a reduction in the number of functional cells [20, 21]. The results of our study also demonstrated that PGI levels decreased as TPOAb levels increased. In the present study, we also observed the slight increase in the PGI/II ratio across TSH level, and we speculated that it mainly was caused by a reduction in PGII, rather than an increase in PGI. Thus, particular attention should be paid to patients with the change of gastric function for the possibility of associated autoimmune thyroid diseases.

Gastrointestinal hormones have function to optimize the process of intestinal digestion and absorption of nutrients [22]. Gastrin normally regulates gastric acid secretion by stimulating the proliferation of enterochromaffin-like cells and the release of histamine [9, 23]. Recently, gastrointestinal hormones play an increasingly important role in the regulation of lipid metabolism. In the research of Saqui-Salces et al. results showed that components of food are sensed by antral cilia on endocrine cells, which can modulate gastrin secretion and gastric acidity [24]. Our present study also found that as serum TG level increased, serum G17 level increased. The above results suggested that, in addition to reflecting abnormal inflammation in the antrum, elevated G17 level also was related to abnormal lipid metabolism in the body.

In accordance with some previous studies, our results showed that the serum PGI/II ratio was positively associated with glucose and HbA1C levels. Tanaka et al. demonstrated that a lack of gastric acid in AG influences the absorption of a variety of nutrients. Sipponen and Härkönen showed that AG may be a risk factor for malabsorption of dietary and supplementary calcium and may therefore increase the risk of osteoporosis on the long term [25]. Tanaka et al. also found that the PG I/II ratio, which was associated with AG, was an independent determinant of glucose levels. One proposed mechanism suggests that lower glucose levels may be caused by poor absorption during the decline of PGI/II ratio. Our results confirmed a positive correlation between glucose levels and the PGI/II ratio.

There were several limitations to our study. First, although the correlations between serum indicators of gastric and extragastric functions were adjusted by sex, age, and* H. pylori* infection, information about other potential confounding factors such as unhealthy living habits (e.g., smoking and drinking) was lacking from this retrospective study. Second, the present study was designed to investigate the correlations between gastric and various extragastric function parameters but was not able to detect causal links between these indicators. For example, although we found the association between Cr level and PGI and PGII concentrations, no reasonable explanation was given for the possible molecular mechanism because we do not do the mechanism research, which needs to be warranted in the further.

## 5. Conclusions

In conclusion, our results suggest that serum PG and G17 levels were associated with blood glucose and lipids and kidney function thyroid function but not with liver function. Serum indicators reflecting gastric function may correlate not only with primary diseases, but also with other extragastric diseases.

## Figures and Tables

**Table 1:** Baseline characteristics.

Parameters	Median (interquartile range)	Normal range	Minimum and maximum value	Kolmogorov-Smirnov test
sPGI (microg/L)	94.9 (73–119.4)	70–150	13.9, 583.4	0.000
sPGII (microg/L)	7.5 (5.3–12)	≥10.25	1.3, 98.1	0.000
PGI/II	12.06 (8.9–15.45)	≥7	1.52, 53	0.023
sG17 (pmol/L)	2.2 (0.9–5.9)	≤5	0.0, 150.1	0.000
Cr (*μ*mol/L)	68 (56–77)	59–104	37, 394	0.000
Ure (mmol/L)	5.18 (4.45–6.1)	2.85–7.14	2.31, 15.28	0.003
ALT (U/L)	21 (15–32)	9–50	6, 765	0.000
ALP (U/L)	67 (56–79)	45–125	30, 208	0.000
FT3 (pmol/L)	4.64 (4.26–5.0)	2.63–5.70	2.79, 39.63	0.000
TSH (mIU/L)	1.63 (1.13–2.37)	0.35–4.94	0.00, 65.58	0.000
TPOAb (IU/mL)	0.29 (0.09–0.78)	0.00–5.61	0.00, 952.8	0.000
TGAb (IU/mL)	1.2 (0.71–2.58)	0.00–4.11	0.00, 908.3	0.000
TG (mmol/L)	1.43 (0.96–2.14)	0.00–1.70	0.31, 32.92	0.000
HDL (mmol/L)	1.23 (1.04–1.46)	0.91–1.92	0.52, 2.91	0.000
LDL (mmol/L)	3.2 ± 0.84	0.00–3.64	0.35, 7.41	0.195
Glu (mmol/L)	5.53 (5.19–6.17)	3.90–6.10	4.07, 18.24	0.000
HbA1C (mmol/mol)	6.68 ± 1.49	3.90–6.10	4.8, 11.9	0.053

**Table 2:** The correlations between gastric function indicators and Cr and urea nitrogen levels in serum.

Gastric function	Cr level quartiles^a^				*P*	Urea level quartiles^b^				*P*
	Q1 (*N* = 209)	Q2 (*N* = 218)	Q3 (*N* = 194)	Q4 (*N* = 202)		Q1 (*N* = 208)	Q2 (*N* = 208)	Q3 (*N* = 203)	Q4 (*N* = 204)	
PGI	79.7 (66.6–109.75)	92.75 (72.3–119.73)	96.65 (75.15–117.55)	105.15 (80.9–129.8)	0.044	86.15 (67.43–110.45)	96.2 (73.4–121.88)	94.4 (72.2–115.7)	99.8 (80.4–129.5)	0.305
PGII	6.5 (4.86–10.75)	7.81 (5.1–12.45)	7.75 (5.5–12.3)	8.4 (5.8–13.23)	0.024	7.2 (5.0–11.7)	7.25 (5.4–11)	7.4 (5.0–13.1)	8.25 (5.73–12.2)	0.160
PGI/II	11.97 (8.52–15.62)	12.03 (8.79–15.14)	12.03 (9.05–15.46)	12.27 (9.27–15.67)	0.047	11.89 (8.47–14.93)	12.67 (9.35–15.86)	11.68 (8.35–15.15)	12.3 (9.1–15.7)	0.993
G17	2.4 (0.98–6.6)	2.45 (0.94–5.73)	1.78 (0.69–5.34)	2.18 (0.8–6.63)	0.101	1.65 (0.85–5.64)	2.05 (0.78–5.2)	2.9 (0.8–7.6)	2.55 (1.0–5.9)	0.416

Cr: creatinine.

^a^Quartile 1 (37–56 *μ*mol/L); quartile 2 (57–68 *μ*mol/L); quartile 3 (69–77 *μ*mol/L); quartile 4 (78–394 *μ*mol/L).

^b^Quartile 1 (2.31–4.45 mmol/L); quartile 2 (4.46–5.18 mmol/L); quartile 3 (5.19–6.10 mmol/L); quartile 4 (6.11–15.28 mmol/L).

The values listed in the table are median and interquartile range.

**Table 3:** The correlation between gastric function indicators and ALT and ALP levels in serum.

Gastric function	ALT level quartiles^a^				*P*	ALP level quartiles^b^				*P*
	Q1 (*N* = 206)	Q2 (*N* = 232)	Q3 (*N* = 182)	Q4 (*N* = 203)		Q1 (*N* = 215)	Q2 (*N* = 208)	Q3 (*N* = 198)	Q4 (*N* = 202)	
PGI	89.7 (69.9–115.85)	97.95 (76.08–121.7)	96.2 (72.28–121.7)	95.3 (72.3–119.4)	0.923	89 (70.3–118.5)	95.95 (77.05–116.48)	96.7 (73–123.85)	95.5 (73.65–122.88)	0.397
PGII	7.25 (5.18–13.03)	7.6 (5.53–11.53)	7.21 (5.18–12.13)	7.8 (5.1–12)	0.640	7.3 (5.0–12.5)	7.2 (5.3–11)	8.05 (5.58–12.6)	7.6 (5.3–12.03)	0.331
PGI/II	11.69 (8.01–15.29)	12.41 (9.56–15.7)	12.17 (8.59–15.05)	12.1 (8.9–15.64)	0.639	12.04 (8.57–15.69)	12.92 (9.13–15.67)	11.64 (8.76–15.37)	11.98 (8.9–15.3)	0.947
G17	2.28 (0.9–6.63)	2.35 (0.86–5.55)	2.35 (0.83–6.14)	1.9 (0.9–5.4)	0.267	2.1 (0.6–5.7)	1.65 (0.8–5.38)	2.6 (1.14–6.36)	2.53 (0.99–6.31)	0.146

ALT: aminotransferase; ALP: alkaline phosphatase.

^a^Quartile 1 (6–15 U/L); quartile 2 (16–22 U/L); quartile 3 (23–32 U/L); quartile 4 (33–765 U/L).

^b^Quartile 1 (30–56 U/L); quartile 2 (57–67 U/L); quartile 3 (68–79 U/L); quartile 4 (80–208 U/L).

The values listed in the table are median and interquartile range.

**(a):** 

Gastric function	T3 level quartiles^a^				*P*
	Q1 (*N* = 207)	Q2 (*N* = 210)	Q3 (*N* = 204)	Q4 (*N* = 202)	
PGI	94.2 (72.2–121.9)	92.5 (71.23–112)	94.9 (75.55–122.45)	96.15 (72.4–119.95)	0.399
PGII	7.2 (5.3–13.1)	7 (5–10.77)	8.15 (5.63–12.9)	8.15 (5.2–12.23)	0.629
PGI/II	12.15 (8.73–16.08)	12.7 (9.39–15.89)	11.47 (8.83–14.59)	12.01 (8.74–15.52)	0.094
G17	2.6 (0.9–5.7)	2.08 (0.9–5.26)	2.3 (0.81–7.7)	1.95 (0.9–6.05)	0.151

Gastric function	T4 level quartiles^b^				*P*
	Q1 (*N* = 207)	Q2 (*N* = 205)	Q3 (*N* = 207)	Q4 (*N* = 204)	

PGI	96.1 (73–119.8)	91.8 (73–113.3)	92.7 (72.9–117.7)	98.85 (72.78–127)	0.506
PGII	7.6 (5.2–12.3)	6.7 (5.25–11.1)	7.7 (5.3–12.2)	7.96 (5.5–12.55)	0.542
PGI/II	12.2 (8.9–15.31)	12.38 (9.44–15.59)	12.31 (8.36–15.61)	11.82 (8.64–15.55)	0.820
G17	2.55 (0.8–5.9)	2.1 (0.9–5.08)	2.1 (0.8–5.8)	2.28 (0.95–7.6)	0.059

T3: triiodothyronine; T4: tetraiodothyroxide.

^a^Quartile 1 (2.79–4.26 pmol/L); quartile 2 (4.27–4.64 pmol/L); quartile 3 (4.65–5.00 pmol/L); quartile 4 (5.01–39.63 pmol/L).

^b^Quartile 1 (7.31–13.22 U/L); quartile 2 (13.23–14.28 U/L); quartile 3 (14.29–15.44 U/L); quartile 4 (15.45–40.89 U/L).

The values listed in the table are median and interquartile range.

**(b):** 

Gastric function	TSH level quartiles^c^				*P*
	Q1 (*N* = 206)	Q2 (*N* = 210)	Q3 (*N* = 204)	Q4 (*N* = 203)	
PGI	98.35 (75.65–122)	95.05 (72.8–117.9)	91.7 (71.68–122.45)	90.8 (71.8–119.4)	0.516
PGII	8.15 (5.68–13.53)	7.8 (5.5–11.4)	7 (5–11.7)	6.9 (5.1–12)	0.093
PGI/II	11.54 (8.38–15.34)	12.05 (9.5–15.1)	12.09 (9.07–15.6)	12.68 (8.93–15.53)	0.047
G17	2.88 (1.09–7.35)	2.7 (1.04–5.3)	1.78 (0.76–6.18)	2 (0.7–5.3)	0.334

Gastric function	TPOAb level quartiles^d^				*P*
	Q1 (*N* = 210)	Q2 (*N* = 205)	Q3 (*N* = 208)	Q4 (*N* = 200)	

PGI	100.85 (75.1–125.78)	93.1 (74.05–118)	96.9 (73.33–124.75)	84 (68.98–111.4)	0.008
PGII	7.8 (5.5–11.9)	7.3 (5.1–12)	8.65 (5.7–14.08)	6.95 (5–10.78)	0.420
PGI/II	12.86 (9.63–15.8)	12.13 (8.97–15.54)	11.08 (8.07–14.49)	12.17 (9.33–15.57)	0.58
G17	2.63 (1.04–6.3)	2.05 (0.73–5.43)	2.58 (1.1–6.75)	1.83 (0.6–5.39)	0.990

Gastric function	TGAb level quartiles^e^				*P*
	Q1 (*N* = 208)	Q2 (*N* = 206)	Q3 (*N* = 205)	Q4 (*N* = 204)	

PGI	92.9 (72.2–121.45)	98.65 (77.08–121.83)	92.4 (74.55–120.85)	91.7 (69.2–118.25)	0.23
PGII	8.4 (5.1–12.38)	7.8 (5.68–12.53)	7.3 (5.15–11.05)	6.8 (5.2–11.68)	0.37
PGI/II	11.88 (8.78–15.02)	12.51 (9.17–15.57)	12.04 (8.92–15.96)	12.09 (8.56–15.29)	0.21
G17	2.98 (1.11–7.28)	2.1 (0.99–5.31)	2.05 (0.8–5.55)	1.83 (0.7–5.35)	0.07

TSH: thyroid stimulating hormone; TPOAb: thyroid peroxidase antibody; TGAb: thyroglobulin antibody.

^c^Quartile 1 (0–1.13 mIU/L); quartile 2 (1.14–1.63 mIU/L); quartile 3 (1.64–2.37 mIU/L); quartile 4 (2.38–65.58 mIU/L).

^d^Quartile 1 (0–0.10 IU/mL); quartile 2 (0.11–0.29 U/mL); quartile 3 (0.30–0.78 U/mL); quartile 4 (0.79–952.8 IU/mL).

^e^Quartile 1 (0–0.7 IU/mL); quartile 2 (0.72–1.20 IU/mL); quartile 3 (1.21–2.58 IU/mL); quartile 4 (2.59–908.3 IU/mL).

**Table 5:** The correlation between gastric function parameters and TG, LDL, and HDL levels in serum.

Gastric function	TG level quartiles^a^				*P*
	Q1 (*N* = 206)	Q2 (*N* = 207)	Q3 (*N* = 205)	Q4 (*N* = 205)	
PGI	89.15 (69.18–113.78)	98 (74–126)	95.3 (73.9–121.85)	96.1 (73.35–123.15)	0.8
PGII	6.7 (4.98–11.63)	7.7 (5.6–13.7)	7.6 (5.35–11.4)	8.3 (5.3–12)	0.75
PGI/II	12.31 (8.86–15.65)	11.81 (8.39–15.3)	12.21 (8.97–15.55)	12.13 (9.37–15.42)	0.48
G17	1.73 (0.78–4.85)	2.1 (0.85–6.7)	2.35 (0.8–6.1)	2.7 (1.1–6.85)	0.04

Gastric function	LDL level quartiles^b^				*P*
	Q1 (*N* = 208)	Q2 (*N* = 205)	Q3 (*N* = 206)	Q4 (*N* = 204)	

PGI	98.25 (75.78–125.53)	89.3 (70.9–119.25)	91.85 (72.18–112.35)	96.55 (72.48–123.4)	0.045
PGII	8.25 (5.73–12.4)	7.22 (5.1–12.25)	7.25 (5.2–11.73)	7.34 (5.13–11.95)	0.165
PGI/II	12.00 (8.91–14.95)	12.3 (8.92–15.82)	11.94 (8.84–15.32)	12.12 (8.92–15.67)	0.29
G17	2.38 (1–5.49)	2.2 (0.83–6.8)	2.05 (0.89–5.71)	2.38 (0.8–6)	0.412

Gastric function	HDL level quartiles^c^				*P*
	Q1 (*N* = 206)	Q2 (*N* = 211)	Q3 (*N* = 205)	Q4 (*N* = 201)	

PGI	95.7 (73.58–123.4)	97.7 (73.7–120.4)	95 (74.05–122.1)	89.7 (69.9–114)	0.57
PGII	7.8 (5.3–11.3)	8.3 (5.2–13.1)	7.1 (5.3–13)	7 (5.1–11.25)	0.92
PGI/II	12.66 (9.9–15.83)	11.63 (8.34–15.33)	12.18 (8.96–15.49)	11.86 (8.54–15.31)	0.170
G17	2.1 (0.94–6.23)	2.55 (0.95–6.6)	2.1 (0.73–6.45)	2.3 (0.85–4.88)	0.13

TG: triglyceride; LDL: low-density lipoprotein, HDL: high-density lipoprotein.

^a^Quartile 1 (0.31–0.96 mmol/L); quartile 2 (0.97–1.42 mmol/L); quartile 3 (1.43–2.12 mmol/L); quartile 4 (2.13–32.92 mmol/L).

^b^Quartile 1 (0.35–2.66 mmol/L); quartile 2 (2.67–3.15 mmol/L); quartile 3 (3.16–3.69 mmol/L); quartile 4 (3.70–7.41 mmol/L).

^c^Quartile 1 (0.52–1.04 mmol/L); quartile 2 (1.05–1.23 mmol/L); quartile 3 (1.24–1.46 mmol/L); quartile 4 (1.47–2.91 mmol/L).

**Table 6:** The correlation between gastric function indicators and Glu and HbA1C levels in serum.

Gastric function	Glu level quartiles^a^				*P*	HbA1C level quartiles^b^				*P*
	Q1 (*N* = 210)	Q2 (*N* = 204)	Q3 (*N* = 206)	Q4 (*N* = 203)		Q1 (*N* = 15)	Q2 (*N* = 14)	Q3 (*N* = 14)	Q4 (*N* = 13)	
PGI	91.95 (72.88–116.5)	95.2 (72.6–116.03)	94.3 (72.63–122.73)	96.1 (73.1–123.8)	0.191	97.1 (87.1–121.6)	103.1 (83.58–137.73)	108 (77.6–137)	86.5 (70.2–127.05)	0.723
PGII	7.0 (5.4–11.45)	7.8 (5.5–12.98)	7.5 (5.3–13.03)	7.38 (5–11.5)	0.125	9.81 (5.89–13.4)	9.66 (6.75–12.52)	7.7 (5.6–13.4)	7.6 (4.9–10.86)	0.153
PGI/II	11.98 (9.35–15.31)	11.75 (8.48–15)	11.98 (8.67–15.33)	12.67 (9.29–16.39)	0.016	9.9 (7.29–13.4)	11.81 (9.62–13.71)	13.02 (11.1–15)	13.54 (12.05–16.22)	0.046
G17	2.05 (0.95–5.4)	2.35 (0.8–6.28)	2.1 (0.8–6.63)	2.4 (0.9–5.5)	0.608	1.85 (0.6–5.9)	3.78 (0.4–5.74)	2.6 (0.5–5.4)	1.9 (0.9–6.2)	0.549

Glu: glucose; HbA1C: glycated hemoglobin.

^a^Quartile 1 (4.07–5.19 mmol/L); quartile 2 (5.20–5.53 mmol/L); quartile 3 (5.54–6.17 mmol/L); quartile 4 (6.18–18.24 mmol/L).

^b^Quartile 1 (4.8–5.6 mmol/L); quartile 2 (5.7–6.1 mmol/L); quartile 3 (6.2–7.2 mmol/L); quartile 4 (7.3–11.9 mmol/L).
